# Discordance between actual and perceived balance ability relates to quality of life and global cognition in a clinical sample of Parkinson patients

**DOI:** 10.1177/1877718X261423310

**Published:** 2026-03-23

**Authors:** Daniel S Peterson, Jason K Longhurst, Franziska Albrecht, Joanna Weller, Jennifer Vasquez, Myassar Zarif, Mark Gudesblatt, Andrew Hooyman

**Affiliations:** 1 Department of Neurobiology, Care Sciences and Society, Karolinska Institutet, Stockholm, Sweden; 2 College of Health Solutions, Arizona State University, Tempe, AZ, USA; 3 Department of Physical Therapy and Athletic Training, Saint Louis University, St. Louis, MO, USA; 4 Women’s Health and Allied Health Professional Theme, Karolinska University Hospital, Stockholm, Sweden; 5 NYU Grossman Long Island School of Medicine, Mineola, NY, USA; 6 Department of Physical Therapy, Chapman University, Irvine, CA, USA

**Keywords:** Parkinson's disease, alignment, discordance, balance, quality of life

## Abstract

**Background:**

Misalignment between actual and perceived balance ability provides relevant information to understand functional deficits and fall risk. However, few studies have provided a continuous quantification of misalignment in neurological populations such as people with Parkinson's disease (PD).

**Objective:**

Determine whether a continuous quantification of misalignment between actual and perceived balance ability, discordance, relates to functional outcomes such as quality of life and cognition.

**Methods:**

Actual (gait velocity), and perceived (Activities of Balance Confidence) balance, cognition (measured via a computer-based cognitive assessment), and mobility-related quality of life were captured in a clinical sample of 95 people with PD. Primary outcomes were quality of life and cognitive domains frequently altered in people with PD (global cognition & executive function). Secondary cognitive domains assessed were attention, memory, visuo-spatial, verbal function, and information processing. Linear and non-linear models assessed the relationship between discordance, quality of life, and cognition.

**Results:**

Discordance related to mobility-related quality of life, such that under-confidence was related to poorer quality of life. Non-linear (quadratic) models were shown to fit the discordance-Global cognition (*p* = 0.02) data better than linear models such that over- and under-confidence related to poorer cognition. Secondary cognitive domains were not robustly related to discordance.

**Conclusions:**

In a clinical sample of people with PD, discordance was related to mobility-related quality of life and global cognition. Global cognition further exhibited a possible non-linear relationship to discordance indicating that over- or under-confidence may relate to poorer cognition. This work underscores the functional relevance of misalignment of actual and balance abilities.

## Plain language summary

Alignment of actual and perceived balance relates to cognition and quality of life in people with PD.

Actual and perceived balance ability are both closely related to functional outcomes such as falls, quality of life, and cognition. Recent work suggests that the alignment between actual and perceived balance may also provide additional relevant information for these functional outcomes. In particular, a recent measure of alignment, called “discordance” assesses whether people's perceived balance is above or below their actual ability levels. This provides a metric of over or under-confidence compared to actual ability. This discordance has been related to falls in people with Parkinson's disease. In this study, we investigated whether “discordance” between actual and perceived balance related to other functional outcomes, namely cognition and quality of life, in a clinical sample of 95 Parkinson's disease patients. We found that individuals with larger discordance (i.e., over-confident) exhibited higher quality of life. A measure of overall or “global” cognition was also related to discordance such that over- and under-confidence related to poorer cognition. These findings suggest that alignment between actual and perceived balance (here measured as discordance) may have functional relevance to outcomes beyond falls, and cognition may be related to both over and under-confidence of balance abilities.

## Introduction

People with Parkinson's disease (PwPD) exhibit progressive mobility deficits, leading to falls, decreased quality of life, and mortality.^1,2^ Many PwPD also exhibit changes to their balance confidence.^3^ Previous work has shown that in PwPD, reduced confidence is related to negative functional outcomes such as falls and quality of life (QOL^4^). However, confidence should be considered in context of actual balance abilities. For example, in individuals with poor mobility, very high balance confidence (i.e., over-confidence) could result in risky behavior that could lead to negative outcomes such as falls and reduced quality of life. Alternatively, low confidence despite high physical ability (i.e., under-confidence) may lead to an unnecessary restriction of both activity and participation. In either case, misalignment or “discordance” between actual and perceived ability could result in negative outcomes, while alignment, or “concordance” between these outcomes could improve the balance between concern and participation.^5,6^

Several previous investigations have described misalignment of actual (physiological) and perceived ability levels and/or fall risk.^5–9^ For example, Delbaere and colleagues (2010) plotted physiological and perceived fall risk in 500 older adults, along with established cutoff values to identify those with high and low risk.^6^ This resulted in individuals falling into four quadrants, two where physiological and perceived fall risks were aligned (either high or low), and two where physiological and perceived fall risk were misaligned. Misaligned quadrants included high perceived risk and low actual risk (termed “anxious”), and high actual risk but low perceived risk (termed “stoic”). Authors noted that negative outcomes were associated with increases in physiological and perceived fall risk, and that falls were common in both misaligned quadrants. Similar results were subsequently found in older adults with cognitive impairments.^7,9^ Finally, one study utilized this quadrant approach to assess actual and perceived function in PwPD.^5^ Like previous reports, analyses showed that individuals with poor actual and perceived function fell most frequently (88%). However, although samples were small in some quadrants, individuals who were over-confident (low actual balance ability and high balance perception), also fell frequently (80% fall rate).

While previous work provides an important framework for establishing alignment across actual and perceived balance, thresholds and ordinal grouping have some challenges, including 1) high reliance on cutoff thresholds to establish “high” and “low” quadrants, 2) varying sample sizes that fall into each quadrant, and 3) ordinal (rather than continuous) subgrouping which reduces statistical power and places individuals with similar values in different groups despite functionally similar characteristics. We recently developed an approach for quantifying misalignment of actual and perceived function (discordance) that provides a continuous quantification of the degree of misalignment, framed as over- or under-confidence.^10,11^ The continuous discordance metric was related to retrospective falls in PwPD.^10^ This finding was replicated in a separate cohort of PD participants, and across a second metric of balance ability, indicating a replicable relationship to a functional metric (fall history).^11^

Together, previous work indicates that discordance between actual and perceived balance may be a meaningful and functionally relevant outcome. However, additional work is necessary to assess this claim. First, determining whether discordance is related to other meaningful outcomes such as quality of life could lead to a better understanding of the relevance of this outcome. Similarly, identifying relationships to outcomes such as cognition could provide preliminary insight into potential causes of discordance. Finally, previous assessments of discordance in PwPD have been collected in a research setting, where participant selection bias may have influenced generalizability of findings. In particular, studies collected in a laboratory setting may not recruit individuals with more severe balance or cognitive deficits or those who are unable or willing to travel for non-clinical services. Expanding analyses to data collected in the course of routine care in a clinical setting is key to establish the deployability and clinical relevance of this metric.

The purpose of the current analysis is to determine the relationship between discordance (between actual and perceived balance ability) and two functionally relevant outcomes: mobility-related QOL and cognition in a clinical sample of PwPD. We hypothesize that QOL and cognition will exhibit an inverted-U shaped relationship with discordance, such that positive or negative discordance would relate to worse QOL and cognition.

## Methods

### Participants

This was a secondary analysis of a cross-sectional, single-site data collection that occurred as part of routine PD care at the NYU Langone South Shore Neurologic Associates clinic in Patchogue, New York. However, none of the data reported have been published previously in whole or part. Participant inclusion criteria were: PD diagnosis (based on standard clinical criteria) from a board-certified neurologist, age greater than 18 years, and ability to speak and comprehend English. Exclusion criteria were: inability to walk, recent surgical or orthopedic procedures, and pregnancy. Informed consent was waived by the Institutional Review Board (IRB) as the digital gait analysis and computerized cognitive testing were performed as part of routine clinical care and the fully de-identified data were retrospectively analyzed. Approval for the retrospective review of de-identified clinical data was obtained from Solutions IRB. The IRB registration number is IORG0007116 and the FWA number is IRB00008523.

### Data collection & outcomes

Participants’ demographic and clinical characteristics (age, gender, and Hoehn & Yahr scale [H&Y]) were first collected. H&Y was assessed by a board certified neurologist. Participants then completed the Activities of Balance Confidence Scale (ABC^12^). This is a 16 question scale that measures how confident individuals are that they can complete mobility tasks without losing their balance, and has been validated in PwPD.^13^ This scale is freely available for use in both clinical and research settings. All participants completed the ABC with pen and paper at the time of digital gait analysis. Then, participants completed a self-selected, “comfortable speed” walk across a 6.1-meter long, 1.2 m wide instrumented walkway (Zeno Walkway Gait Analysis System; Protokinetics LLC, Havertown PA, USA).^14^ Validity and reliability of gait metrics from the instrumented walkway have been previously reported.^15,16^ Participants completed 3 trials walking across the mat, and gait velocity was calculated as the average across the 3 trials. Velocity was extracted from the instrumented mat system. Comfortable footwear was worn for the walking task. Individuals using walking aids during the assessment were removed from the analysis. The walking test was administered by a certified physical therapist. Five individuals utilized a form of walking aid during walking tests. We chose to remove data from these individuals as the type of aid was not consistently recorded, and varying types of aids may reduce interpretability of data.

Cognition was tested in the clinic using the NeuroTrax computer-based cognitive assessment tool: Global Assessment Battery. NeuroTrax system is a battery of computer-based assessments, totaling approximately 45 min, which assesses several cognitive domains: executive function (EF), attention, memory, verbal function, visuospatial, information processing speed, and motor function). It also provides a metric of global cognition (GC), the “global cognitive summary score” which is the average of all cognitive domains tested. The NeuroTrax system has been shown to be useful in identifying executive cognitive deficits specifically in PwPD.^17^ Our a-priori cognitive outcomes of interest were GC and EF. These primary cognitive outcomes were chosen due to the common deficits observed in these outcomes in PwPD.^18^ However, as other cognitive domains have also been shown to be altered in this population, secondary analyses assessed attention, memory, visuospatial, verbal function, and information processing.

Mobility-related QOL was tested via the Neuro-QOL Item Bank v1.0 - Lower Extremity Function (Mobility). This form consists of 19 questions, each scored on a Likert scale from 1–5 in which a higher score corresponded to improved function. This scale has been shown to be appropriate for use in neurological populations including PwPD.^19,20^

All data were collected from participants in the “ON” medication state. For logistical reasons, gait and cognitive testing were not completed on the same day, however effort was taken to complete assessments at similar time of day. The median (IQR) of days between testing was 37 (13, 75), see Table 1 for details.

**Table 1.** Subject characteristics.

Characteristic	N = 95^a^
Age (years)	71.5 ± 8.2
Gender	
F	35 (37%)
M	60 (63%)
Hoehn & Yahr	
1	6 (6.3%)
1.5	1 (1.1%)
2	27 (28%)
2.5	5 (5.3%)
3	48 (51%)
4	8 (8.4%)
Activities of Balance Confidence (%)	63.4 ± 29.0
Gait Velocity (cm/sec)	82.9 ± 27.8
Global Cognitive Score^b^	92.6 ± 13.7
Executive Function Score^b^	90.8 ± 15.9
Missing	1
Attention Score^b^	91.3 ± 19.9
Missing	9
Quality of Life^c^	66.4 ± 19.1
Missing	21
Days between assessments (median [IQR])	37 [13, 75]

^a^ Mean ± SD unless otherwise indicated.

^b^ NeuroTrax.

^c^ NeuroQOL.

Balance discordance was calculated by fitting a linear regression line between ABC scores and gait speed, with gait speed as the independent variable and ABC score as the dependent variable. A linear model has been shown previously to best fit these types of data.^10^ ABC and gait speed were chosen for theoretical and pragmatic reasons. Previous work calculating discordance has used ABC for the perception of balance ability and several outcomes for gait, including the Timed Up and Go (TUG, with several variations including 3 m, 7 m, comfortable pace, and fast as possible), and the Mini-Balance Evaluation Systems Test. Previous work indicated calculation of discordance was robust across these gait metrics.^10,11^ In the current dataset, and as noted above, participants completed straight-line, comfortable speed walking over an instrumented gait-mat. Gait speed was chosen as the outcome to represent “actual” ability given the similarity with previously used values such as TUG, a valid and generalizable predictor of fall risk in PD populations.

After the regression line between ABC and gait speed was generated among the study sample, the fitted intercept and regression coefficient for gait speed were then used to generate predicted ABC given a participant's gait speed based on the following equation:

ActualABC=12.66+(.61*GaitVelocity)

The resultant regression line had an R^2^ = .34. The predicted ABC value was then subtracted from the individual's actual ABC value to generate a discordance score. A positive discordance score (predicted ABC greater than actual ABC) represents over-confidence, and a negative discordance score (predicted ABC less than actual ABC) represents under-confidence.

### Statistics

Statistical analyses were conducted in R RStudio 2025.05.1 + 513 “Mariposa Orchid” Release. The primary aim was to assess the relationship between calculated discordance and mobility-related QOL and cognition. Given that discordance measures the over or under-confidence of mobility compared to actual mobility levels, we predicted that QOL and cognition would exhibit an inverted-U shaped curve, such that negative discordance (under-confidence) and positive discordance (over-confidence) would be related to poorer QOL and cognition.

To test these relationships, we first fit individual linear models for each outcome. The primary independent variable was discordance, with dependent variables including: QOL, GC, and EF. Age, disease severity (measured as H&Y stage) and gender were included as covariates in all models. These covariates were included to adjust for potential confounds as each outcome can vary as a function of age, gender, and disease severity independent of discordance. It is important to note that H&Y scores were modeled as a continuous variable rather than a factor for all analyses. This was done to prevent overall model distortion due to the imbalanced group sizes across all H&Y severity. Although H&Y is an ordinal variable, and changes in a continuous outcome should not be assumed to be linear in accordance with changes in an ordinal variable, for the range of severity and imbalance across severity it was maintained as a continuous variable to avoid model bias. Quadratic models of the form [y ∼ x + x^2 + age + H&Y + gender] were also explored and statistically compared to linear models to determine which best fit the data. ANOVAs were chosen to compare models as they provide statistical inference tests and p-values to determine whether quadratic models resulted in a statistically significantly improved fit versus a simple linear model. A quadratic model (rather than other non-linear modelling methods, such as segmented regression), was chosen to assess the hypothesized inverted-U relationship because changes in outcomes as a function of discordance were hypothesized to be gradual. Furthermore, fitting of a segmented regression would require validation of a specific known pivot point, which may be interpreted as a value of discordance would be considered optimal or prescriptive in nature, which is a conclusion these data cannot determine. For primary outcomes, high-leverage points were identified via Cooks Distance calculations. Data points with high-leverage were determined by a Cook's distance greater than 4/N (N = number of observations in the model). This yielded a conservative cutoff of approximately 0.04 across variables. For reference, a commonly chosen Cooks distance of 0.5 would have yielded no outliers in the current approach. The more conservative cutoff was chosen due to the moderate sample size available for this study. Each model was run with and without the identified high-leverage points to examine the robustness of fit independent of influential points.

Missing data was handled via a complete-case approach. In instances where >10% of data were missing for a particular variable, sensitivity analyses were run to assess whether missing cases exhibited statistically significant characteristics.

## Results

**Figure 1.**
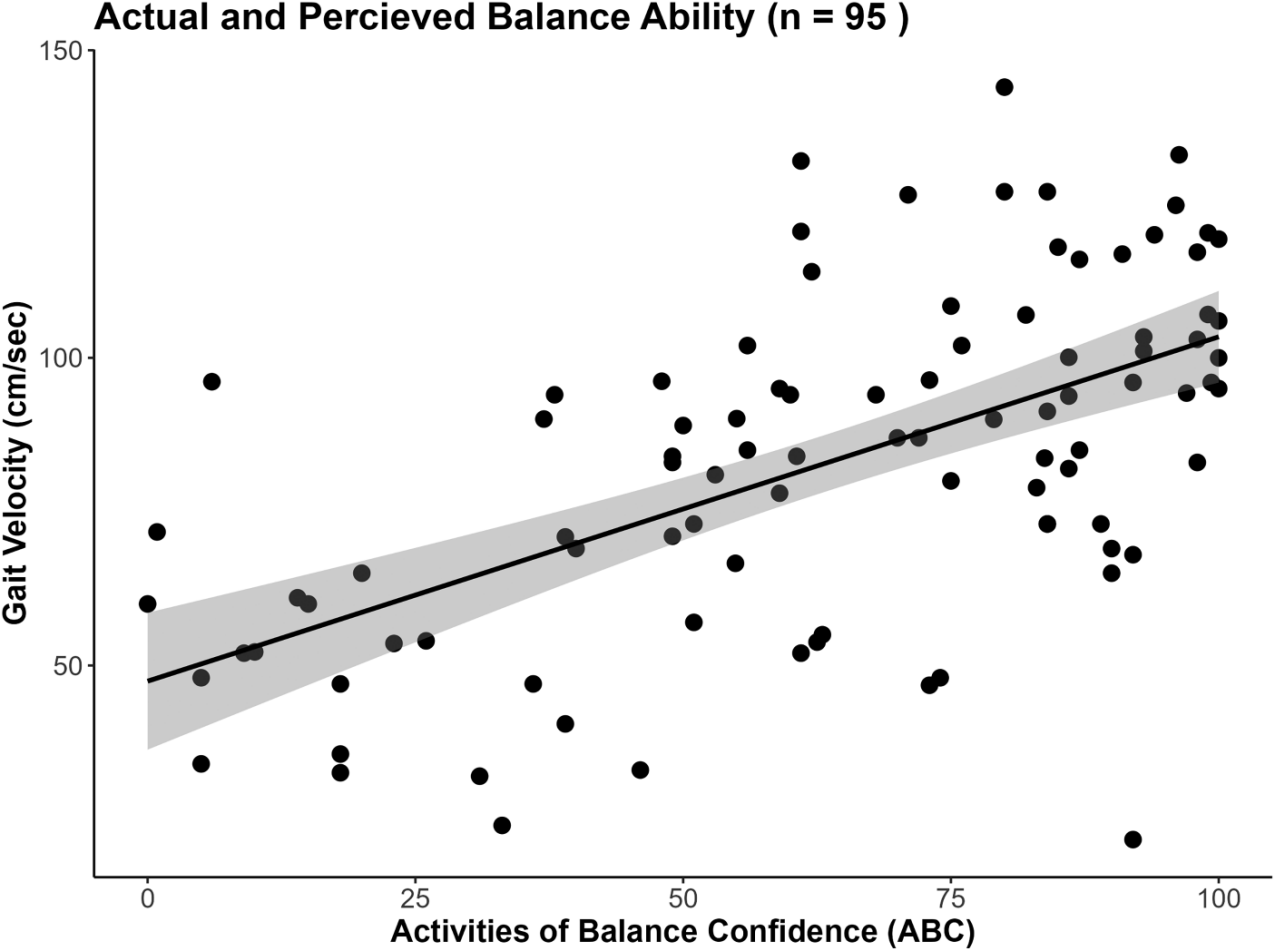
Scatterplot with regression showing the relationship between components of discordance: gait velocity and activities of balance confidence.

Data were available from 95 PwPD (mean age 71.5 (sd = 8.2), female = 35 (37%)) (Table 1). This cohort of PwPD exhibited a range of disability, with a relatively large proportion in the moderate to severe PD stage (H&Y 3 or 4 = 54 (59.4%)). Data were missing from EF (missing = 1) and QOL (missing = 21) cognitive outcomes, see Table 1. There were no statistically significant differences in any outcomes between participants who were or were not missing QOL data (see Supplemental Table 5). Figure 1 shows the scatterplot of gait velocity vs. activities of balance confidence, along with the regression line fit to these data, upon which discordance was calculated.

### The relationship between discordance and mobility-related QOL, global cognition (GC), & executive function (EF)

Linear regression analyses evaluated the association between balance discordance (z-scored) and three primary outcomes: mobility-related QOL, GC, and EF, adjusting for age, Hoehn & Yahr stage, and gender. The z-score was achieved by the “scale” function in R, which takes the difference of each datapoint from the mean, then divides by the standard deviation of the dataset. Data are visualized in Figure 1, and full model details are reported in Table 2.

**Table 2.** Linear regression models of discordance predicting functional outcomes.

	Quality of life			Global cognition			Executive function		
Characteristic	Beta	95% CI	*p*-value	Beta	95% CI	*p*-value	Beta	95% CI	*p*-value
(Intercept)	106	72, 140	<0.001	115	92, 139	<0.001	102	75, 130	<0.001
Discordance (z-score)	6.8	2.9, 11	<0.001	4.0	1.4, 6.7	0.003	4.1	0.99, 7.1	0.010
Age (years)	−0.08	−0.59, 0.42	0.7	−0.18	−0.51, 0.16	0.3	0.04	−0.36, 0.44	0.8
Gender									
F	—	—		—	—		—	—	
M	−1.7	−9.2, 5.8	0.7	2.4	−3.1, 7.9	0.4	4.3	−2.1, 11	0.2
Hoehn & Yahr	−12	−18, −6.8	<0.001	−4.4	−8.2, −0.52	0.026	−6.5	−11, −1.9	0.006

CI: confidence interval.

**Figure 2.**
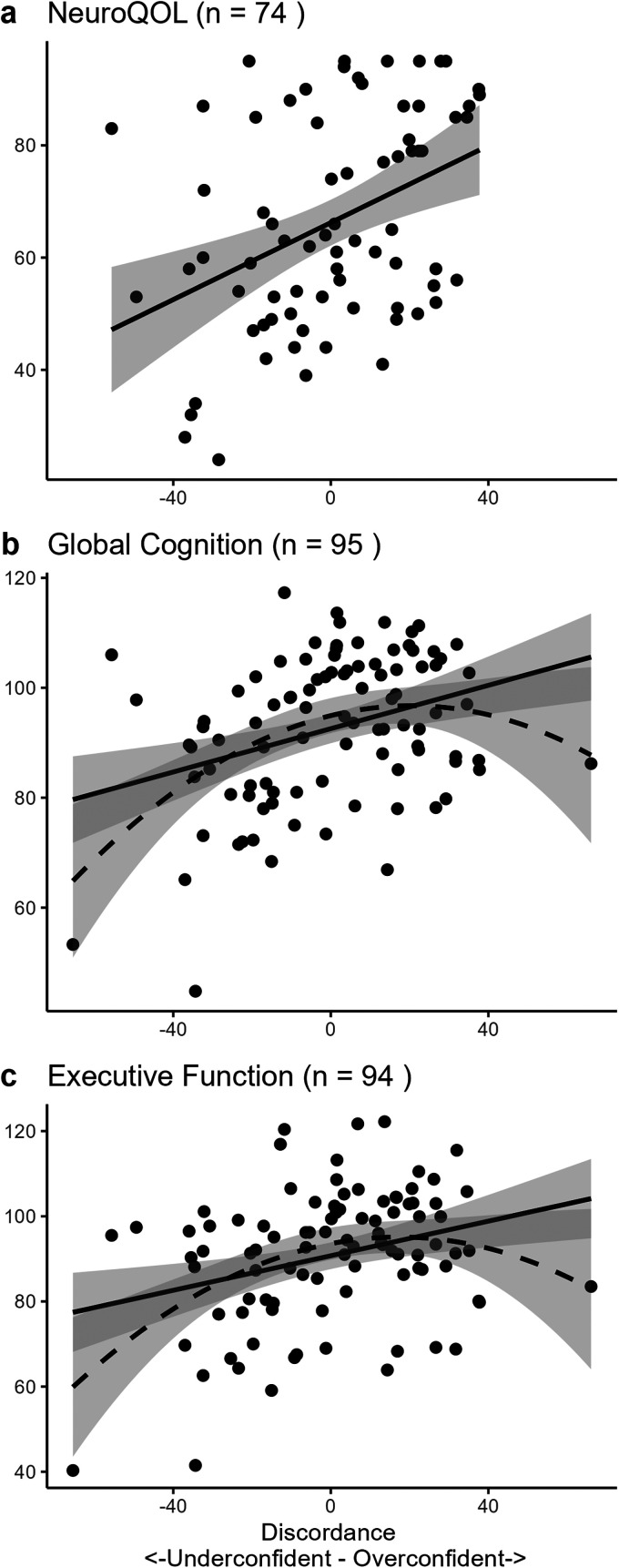
Scatterplots showing relationships between discordance and primary outcomes: (a) mobility-related quality of life, (b) global cognition, and (c) executive function. Non-linear relationships are shown as a dotted line for global cognition and executive function, in which non-linear relationships fit data significantly better than linear relationships.

Linear models indicated that discordance was significantly positively related to each primary outcome, such that positive discordance (i.e., over-confidence) was related to higher self-reported QOL, and negative discordance (i.e., under-confidence), was related to lower self-reported QOL (β_discordance_ = 6.8, 95% CI = [2.9, 11.0], *p* = 0.001; Figure 2(a)). Positive, linear relationships were also observed for GC and EF (GC: β_discordance_ = 4.0, 95% CI = [1.4, 6.7], *p* = 0.003; EF: β_discordance_ = 4.1, 95% CI = [1.0, 7.1], *p* = 0.010; Figure 2(b) and (c)), however as noted below, quadratic models fit these data better. For each model, H&Y staging was significantly negatively correlated to discordance, such that those with more severe PD had worse discordance. Neither age nor gender were significant predictors across the 3 models.

### Comparison of linear and quadratic models of discordance

When linear models were compared to their corresponding quadratic model, results from analysis of variance demonstrated that quadratic models were a better fit than a simple linear model for GC (*p* = 0.03) and EF, (*p* = 0.02), but not QOL (*p* = 0.53; Table 3). This suggests a potential non-linear relationship between cognition and discordance, such that over *or* under-confident (designated by positive or negative discordance values) were related to lower cognition but not QOL. Further review of the quadratic terms in each of the cognitive models demonstrated that the shape of the non-linear relationship reflected that of an inverted-U, as hypothesized (see Figure 2).

**Table 3.** Linear and non-linear models of primary outcome measures (quality of life, global cognition, and executive function).

Model	QOL model	GC model	EF model
Linear r2	0.356	0.158	0.140
Quadratic r2	0.351	0.193	0.182
*P*-value	0.506	0.030	0.020

QOL: quality of life; GC: global cognition; EF: executive function.

To determine if the quadratic relationship observed between discordance and GC & EFis distinct from raw ABC score, we also generated linear and quadratic models with ABC as the primary independent variable in substitution for discordance. When the corresponding linear and quadratic models for each outcome with ABC were compared via analysis of variance there was no significant difference for either GC scores (*p* = .29) or for EF (*p* = .29). This result suggests that discordance possesses a unique non-linear relationship to cognition whereas raw ABC scores does not. Figures for each ABC model for each outcome can be viewed in Supplemental Figure 3.

### Robustness of linear and non-linear relationships with removal of high-leverage datapoints

High leverage datapoints were noted for QOL, GC, and EF models (n = 3, 4, and 7, respectively; highlighted in red or blue in Supplemental Figure 1). As such, all models were re-run with these points removed. For the linear models, QOL (*p* < .001) and GC (*p* = .009) remained significantly related to discordance, but the relationship to EF lost significance (*p* = 0.2, Supplemental Table 1). Additionally, in these high-leverage data removed datasets, the quadratic model for GC remained statistically superior to the linear model for GC (*p* = 0.006), but not EF (*p* = 0.06; Supplemental Table 2). The lines of best fit for each outcome after removal of high leverage points are represented by the red or blue dashed line in Supplemental Figure 2).

### Relating secondary cognitive values and discordance

Of the 6 secondary cognitive variables tested (attention, memory, visuospatial, verbal function, information processing, and motor function), only visuospatial function was statistically significantly related to discordance (β_discordance_ = 0.4, 95% CI = [1; 7.9], *p* = 0.01); Supplemental Table 3; Supplemental Figure 2). Non-linear relationships were not robustly observed in these outcomes with only verbal fluency demonstrating a better fit with quadratic versus linear discordance terms. However, the overall explanatory power of this model was low with an R-squared of 0.038 (Supplemental Table 4).

## Discussion

The purpose of this study was to determine whether a continuous measure of misalignment between actual and perceived balance ability (discordance) is related to functional outcomes including mobility-related QOL (measured via the NeuroQOL Lower Extremity Function [Mobility] subscore) and cognition (measured via the NeuroTrax cognitive battery). In partial support of our hypothesis, we observed discordance to relate to mobility-related QOL and two metrics of cognition (GC and EF, areas particularly impacted in PwPD^1^). We also observed GC and EF (but not mobility-related QOL) to exhibit a statistically significant non-linear relationship to discordance such that negative or positive discordance (over and under-confidence, respectively) may relate to reduced cognitive outcomes. Interestingly, non-linear models relating ABC to cognition were *not* superior to simple linear models. This suggests that discordance (which frames confidence as “over and under” rather than high vs. low, as in ABC) may be uniquely non-linearly related to some cognitive outcomes. Finally, mobility-related QOL was linearly related to discordance, such that increasing values of discordance related to better functional outcomes even when people move into an over-confident discordant state. Together, these findings extend previous work by 1) evaluating a clinical sample of PwPD, 2) relating discordance to additional functional outcomes beyond mobility, and 3) utilizing gait speed, rather than previously used TUG time or the Mini Balance Evaluations Systems Test^10,11^ to calculate discordance.

We observed a linear relationship between discordance and mobility-related QOL. This relationship was robust after a highly conservative removal of high leverage datapoints. In general, this finding extends previous work suggesting that discordance is related to functionally relevant mobility outcomes (falls)^10,11^ to indicate that discordance may also relate to non-mobility outcomes. The data did not support a non-linear, or inverted-u shaped relationship between discordance and mobility-related QOL. This is somewhat surprising, as it is plausible that over-confidence could relate to negative mobility outcomes, thus negatively influencing QOL. However, previous work suggests that other mobility outcomes, such as falls, also showed linear relationships to discordance, such that higher confidence related to lower incidence of fall history.^10,11^ Further, it is possible that increased balance confidence (even confidence that exceeds actual ability levels) could be related to a more positive outlook on life and thus increased QOL. Further, these findings are partially consistent with studies using quadrant-based mis-alignment approaches.^6,8^ For example, Delbaere et al. 2010 showed that QOL was worse in individuals who with lower confidence, regardless of their actual ability levels (either low or high physiological fall risk^6^). Together, these results indicate that lower or under-confidence (quantified by either absolute or relative [i.e., discordance] methods), relates to reduced QOL.

We also observed a relationship between discordance and GC and EF. Further, a quadratic model between discordance and GC and EF better fit data than a linear model, such that either over or under-confidence (denoted by positive or negative discordance), related to reduced cognition. Notably, with a conservative removal of high-leverage datapoints, the quadratic model remained superior for GC, while the EF model lost statistical significance (*p* = 0.06). Further, inspection of Figure 2 shows the somewhat subtle nature of this non-linear relationship in both cognition metrics. These caveats aside, the non-linear relationship between discordance and cognition is partially consistent with previous work. For example, two recent studies indicated that in older adults with mild-moderate cognitive impairment, overconfident participants (i.e., those that were “unaware” of high physiological fall risk) had poorer cognition than other quadrants.^7,9^ Together, these relationships suggest that lower cognition relates to misalignment of actual and perceived ability. This indicates that discordance may provide unique information beyond balance confidence (e.g., ABC) alone. The rationale for a non-linear relationship between cognition and discordance (but not balance confidence) is not entirely clear, and additional work will be necessary to confirm these findings. Further, given the cross-sectional nature of the current dataset, the nature (causal vs. corollary) and direction of these relationships is unknown. Larger cohorts with either directional analyses (e.g., mediation) or longitudinal and/or interventional designs are necessary to understand these links. Further, of the 5 secondary cognitive variables tested, only visuospatial was related to discordance.

Relationships between discordance and secondary cognitive outcomes (attention, memory, visuospatial, verbal function, and information processing) were less robust, as the only statistically significant relationship was observed with visuospatial function. The rationale for a lack of robust relationships across all cognitive domains is unknown, and a clearer understanding of the directionality and nature of these relationships is necessary for effective speculation. It is however interesting that along with GC and EF, visuospatial function is also commonly impacted in PwPD.^18^ It therefore is possible that a larger dynamic range of GC, EF, and visuospatial function in the PD cohort may have facilitated the observed significant relationships to discordance.

To date, few studies have investigated the relationship between misalignment and cognition in individuals with movement disorders (e.g., multiple sclerosis, PD, etc.). Zanotto et al. (2024) showed no statistically significant relationship between cognition (also measured via NeuroTrax) and balance misalignment in people with multiple sclerosis.^21^ However, there are several differences across studies that may explain this discrepancy. First, Zanotto and colleagues investigated multiple sclerosis, which has a somewhat different profile of cognitive dysfunction than PD.^22^ Further the approach to quantify discordance utilized quadrants rather than using a continuous scale of over and under-confidence. Additional work across populations will be necessary to understand the relationship between actual/perceived mobility alignment and cognition. Nonetheless, these initial findings indicate a relationship may exist (at least in PwPD), and this relationship may be linear or non-linear in nature.

We propose that measures of misalignment may provide relevant information to optimize treatment in a patient centric manner. For example, as can be seen in Figure 2, there are individuals with low mobility-related QOL who exhibit positive and negative discordance scores. Knowing where within the mobility-related QOL and discordance space individuals lie may help personalize treatment approaches. It is also notable that the non-linear relationship between GC and discordance may also present a unique treatment challenge. Specifically, treatments focused on changing the perceptual component of discordance, i.e., balance confidence, through a falls-efficacy^23^ or capability-based^24^ approach may require a nuanced approach depending on which side of the non-linear relationship a patient presents. Notably however, additional work is necessary to identify which types of interventions are most effective for individuals across the physiological and perception deficit space.

Several strengths and limitations should be noted. First, this sample was collected in a clinical setting. This allows assessment of a more representative population of PwPD, reduces the likelihood of selection bias, and confirms the ability to collect data to calculate discordance. However, collection in this fashion has drawbacks, including less ability to collect other datapoints such as motor function (e.g., MDS-UPDRS). Second, despite a moderate sample size with broad representation, we observed relatively few individuals in misaligned quadrants, including 7 individuals with relatively low ABC but high (>100 cm/s) gait velocity. The continuous discordance assessment increases our power to calculate misalignment despite small numbers in these misaligned quadrants, however in general, increasing samples in this data space could improve power to understand predictors and treatments for these individuals. Third, while we do have some cognitive metrics, we do not have diagnoses of mild cognitive impairment or similar functional cognitive decline, reducing ability to subtype participants. Fourth, we do not have timed up and go data to conduct a head to head evaluation of discordance using this metric (as has been done previously^10,11^). However, previous results have shown discordance to be robust to different balance metrics,^11^ somewhat reducing this concern. Fifth, cognitive and gait data were often not collected on the same day. This may have added additional noise to our data, reducing the strength of the observed relationships, however it is unlikely to reduce the interpretability of findings. Sixth, we applied a conservative removal of high-leverage datapoints, which reduced the statistical significance the linear and quadratic EF – discordance relationships. As such, the EF - discordance findings should be treated with caution. Seventh, as noted in the methods, we treated Hoehn & Yahr staging (a covariate in analyses) as a continuous variable due to the variance and uneven distribution of this outcome across participants. While we feel this was scientifically justified (see methods), it may have had a minor impact on the overall fit of the models. Finally, given the cross-sectional nature of these data, it is not possible to make causal inferences across variables.

## Conclusion

This study reinforces the functional importance of the alignment between actual and perceived balance ability in PwPD. Using a continuous measure of discordance, we observed that both under and over-confidence were associated with poorer GC, while under-confidence alone was linked to reduced mobility-related QOL. These findings extend prior work by demonstrating that discordance not only relates to fall risk but also to broader aspects of functioning, including cognition and well-being, in a clinically representative sample. Future work should investigate the nature (i.e., direction and causality) of these relationships, whether interventions targeting perceptual-motor alignment can improve mobility-related QOL or cognitive performance, and whether discordance might serve as a clinical marker for tailoring rehabilitative strategies. Taken together, these findings suggest that addressing the alignment between balance perception and ability could represent a novel and impactful direction in rehabilitation care for PwPD.

## Supplemental Material

sj-docx-1-pkn-10.1177_1877718X261423310 - Supplemental material for Discordance between actual and perceived balance ability relates to quality of life and global cognition in a clinical sample of Parkinson patients Supplemental material, sj-docx-1-pkn-10.1177_1877718X261423310 for Discordance between actual and perceived balance ability relates to quality of life and global cognition in a clinical sample of Parkinson patients by Daniel S Peterson, Jason K Longhurst, Franziska Albrecht, Joanna Weller, Jennifer Vasquez, Myassar Zarif, Mark Gudesblatt and Andrew Hooyman in Journal of Parkinson's Disease
