# Supplementary material for: Discordance between actual and perceived balance ability relates to quality of life and global cognition in a clinical sample of Parkinson patients
Source: J Parkinsons Dis. 2026 Mar 23;16(3):529–38. doi: 10.1177/1877718X261423310 (PMC13347598; doi:10.1177/1877718X261423310)
Supplement: sj-docx-1-pkn-10.1177_1877718X261423310 - Supplemental material for Discordance between actual and perceived balance ability relates to quality of life and global cognition in a clinical sample of Parkinson patients [file sj-docx-1-pkn-10.1177_1877718X261423310.docx]

**SUPPLEMENTAL MATERIALS**

**Supplemental Figure 1**: Scatterplots of primary outcome measures (quality of life, global cognition, and executive function) for linear models (left column, panels a, c, and e) and quadratic models (right column, panels b, d, and f). Outliers (circled in red or blue for linear and non-linear models, respectively) are shown. Dotted lines show regression lines after removal of outliers. For both linear and non-linear models, the executive function lost significance after outlier removal (see also Table 1, Supplemental table 1, and Supplemental Table 2).


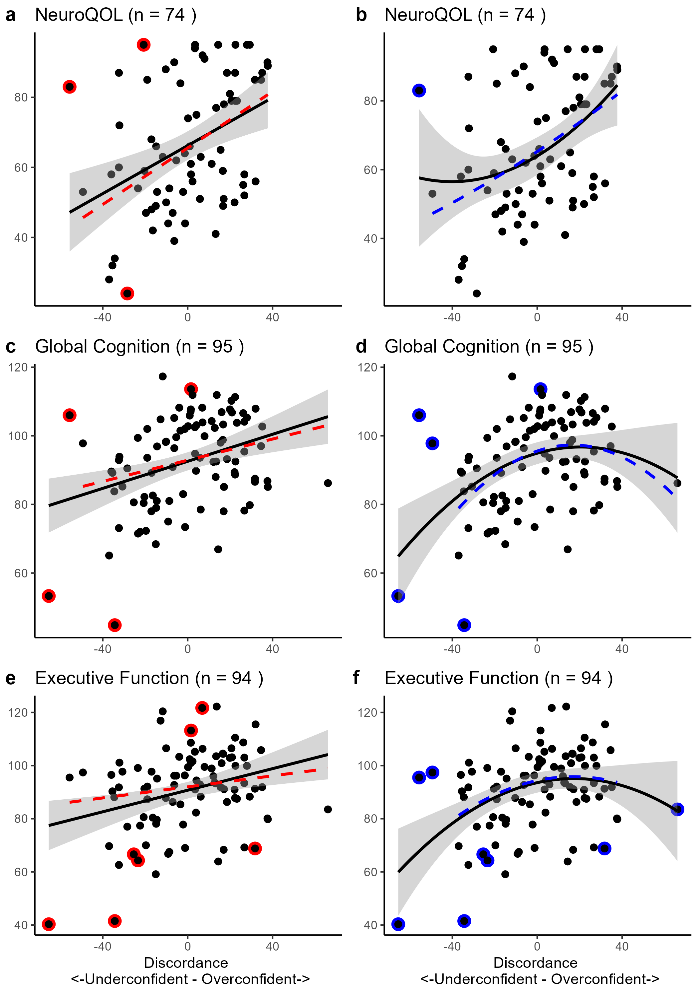


**Supplemental Figure 2**: Scatterplots showing relationships between discordance and secondary cognitive outcomes


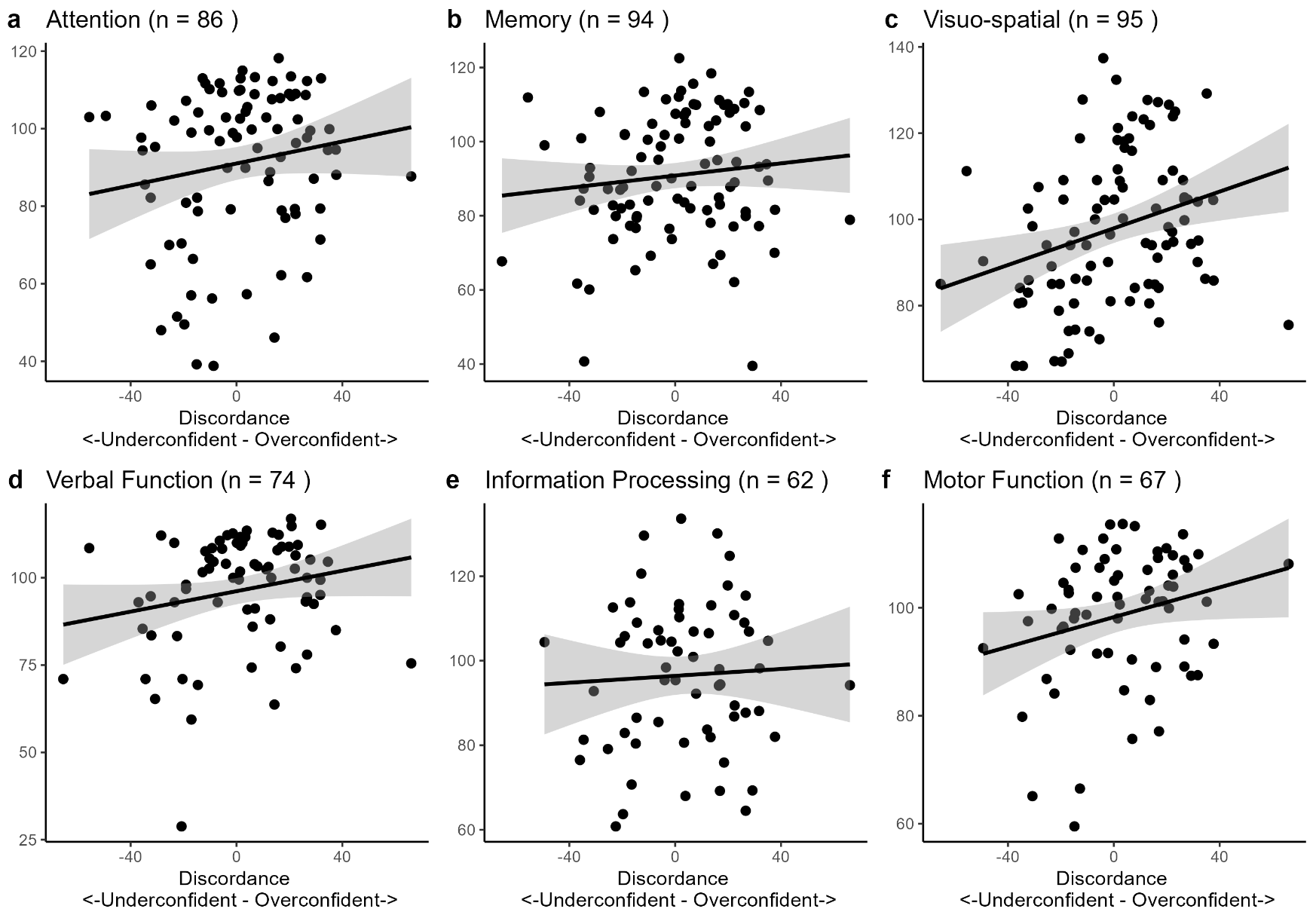


***Supplemental Table 1****. Linear Regression Models of Discordance Predicting functional outcomes- Outliers removed*

|  | Model 1; QOL | | | Model 2; GC | | | Model 3; EF | | |
| --- | --- | --- | --- | --- | --- | --- | --- | --- | --- |
| Characteristic | Beta | 95% CI^1^ | p-value | Beta | 95% CI^1^ | p-value | Beta | 95% CI^1^ | p-value |
| (Intercept) | 106 | 73, 138 | <0.001 | 118 | 96, 141 | <0.001 | 112 | 85, 139 | <0.001 |
| Discordance (z-score) | 8.1 | 4.4, 12 | <0.001 |  |  |  |  |  |  |
| Age (years) | -0.10 | -0.57, 0.38 | 0.7 |  |  |  |  |  |  |
| Gender |  |  |  |  |  |  |  |  |  |
| F | — | — |  |  |  |  |  |  |  |
| M | -3.2 | -10, 3.9 | 0.4 |  |  |  |  |  |  |
| Hoehn & Yahr | -12 | -17, -6.5 | <0.001 |  |  |  |  |  |  |
| Discordance (z-score) |  |  |  | 3.3 | 0.86, 5.7 | 0.009 |  |  |  |
| Age (years) |  |  |  | -0.26 | -0.58, 0.06 | 0.11 |  |  |  |
| Gender |  |  |  |  |  |  |  |  |  |
| F |  |  |  | — | — |  |  |  |  |
| M |  |  |  | 0.46 | -4.7, 5.6 | 0.9 |  |  |  |
| Hoehn & Yahr |  |  |  | -2.7 | -6.3, 0.94 | 0.15 |  |  |  |
| Discordance (z-score) |  |  |  |  |  |  | 2.0 | -0.83, 4.8 | 0.2 |
| Age (years) |  |  |  |  |  |  | -0.12 | -0.51, 0.28 | 0.6 |
| Gender |  |  |  |  |  |  |  |  |  |
| F |  |  |  |  |  |  | — | — |  |
| M |  |  |  |  |  |  | 1.6 | -4.5, 7.7 | 0.6 |
| Hoehn & Yahr |  |  |  |  |  |  | -4.8 | -9.2, -0.44 | 0.031 |
| ^1^CI = Confidence Interval | | | | | | | | | |

| **Supplemental Table 2: Linear and non-linear models of primary outcome measures (QOL, GC, and EF) without high-leverage points** | | | |
| --- | --- | --- | --- |
| Model | QOL model | GC model | EF model |
| Linear r2 | 0.417 | 0.111 | 0.078 |
| Quadratic r2 | 0.414 | 0.179 | 0.107 |
| P-value | 0.412 | 0.006 | 0.061 |

***Supplemental Table 3.*** *Linear Regression Models of Discordance Predicting Secondary Cognitive Outcomes*

|  | **Attention** | | | **Memory** | | | **Visuo-spatial** | | | **Verbal Function** | | | **Info-Processing** | | | **Motor Function** | | | |
| --- | --- | --- | --- | --- | --- | --- | --- | --- | --- | --- | --- | --- | --- | --- | --- | --- | --- | --- | --- |
| **Characteristic** | **Beta** | **95% CI** | **p-value** | **Beta** | **95% CI** | **p-value** | **Beta** | **95% CI** | **p-value** | **Beta** | **95% CI** | **p-value** | **Beta** | **95% CI** | **p-value** | **Beta** | **95% CI** | **p-value** |  |
| (Intercept) | 95 | 54, 136 | <0.001 | 108 | 77, 139 | <0.001 | 114 | 83, 145 | <0.001 | 103 | 70, 136 | <0.001 | 133 | 93, 172 | <0.001 | 82 | 55, 108 | <0.001 |  |
| Discordance (z-score) | 2.9 | -1.5, 7.3 | 0.2 | 1.6 | -1.9, 5.1 | 0.4 | 4.4 | 0.98, 7.9 | 0.012 | 3.3 | -0.68, 7.2 | 0.10 | 0.49 | -4.5, 5.5 | 0.8 | 3.0 | -0.32, 6.2 | 0.076 |  |
| Age (years) | 0.12 | -0.45, 0.69 | 0.7 | -0.14 | -0.59, 0.30 | 0.5 | -0.10 | -0.55, 0.34 | 0.6 | -0.06 | -0.53, 0.42 | 0.8 | -0.34 | -0.90, 0.23 | 0.2 | 0.10 | -0.28, 0.48 | 0.6 |  |
| Gender |  |  |  |  |  |  |  |  |  |  |  |  |  |  |  |  |  |  |  |
| F | — | — |  | — | — |  | — | — |  | — | — |  | — | — |  | — | — |  |  |
| M | 5.4 | -3.6, 14 | 0.2 | 0.35 | -6.9, 7.6 | >0.9 | 3.9 | -3.3, 11 | 0.3 | -0.10 | -8.2, 8.0 | >0.9 | 1.7 | -7.9, 11 | 0.7 | 4.0 | -2.4, 10 | 0.2 |  |
| Hoehn & Yahr | -6.0 | -13, 0.53 | 0.071 | -2.7 | -7.8, 2.4 | 0.3 | -4.3 | -9.3, 0.77 | 0.10 | -0.89 | -6.5, 4.7 | 0.8 | -5.4 | -13, 2.1 | 0.2 | 2.7 | -2.5, 7.8 | 0.3 |  |
| Abbreviation: CI = Confidence Interval | | | | | | | | | | | | | | | | | | | |

| **Supplemental Table 4:** Linear and non-linear models of secondary cognitive outcome measures | | | | | | | |
| --- | --- | --- | --- | --- | --- | --- | --- |
| Model | Attention Model | Memory Model | Visuo-spatial Model | Verbal function Model | Information Processing Model | Motor Function Model |  |
| Linear r2 | 0.027 | -0.0 | 0.090 | -0.009 | 0.015 | 0.059 |  |
| Quadratic r2 | 0.015 | 0.0 | 0.107 | 0.038 | 0.011 | 0.058 |  |
| P-value | 0.786 | 0.1 | 0.100 | 0.040 | 0.376 | 0.336 |  |

Supplemental Figure 3: Linear and non-linear relationships between ABC and global cognition (left) and executive function (right). The lack of non-linear relationships between ABC and these outcomes (along with the statistically significant non-linear relationship to discordance indicates that discordance may be unique in its non-linear relationship to these cognitive outcomes.


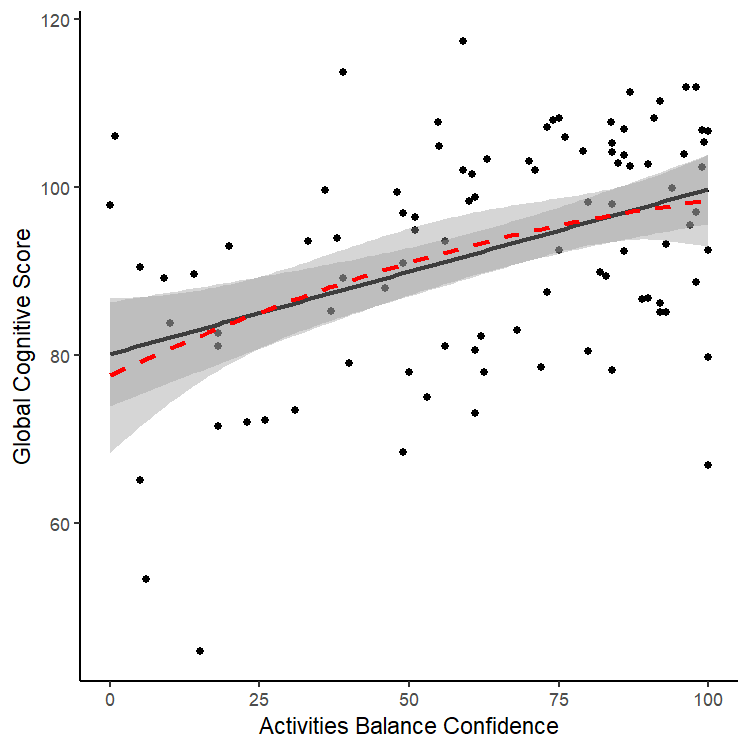

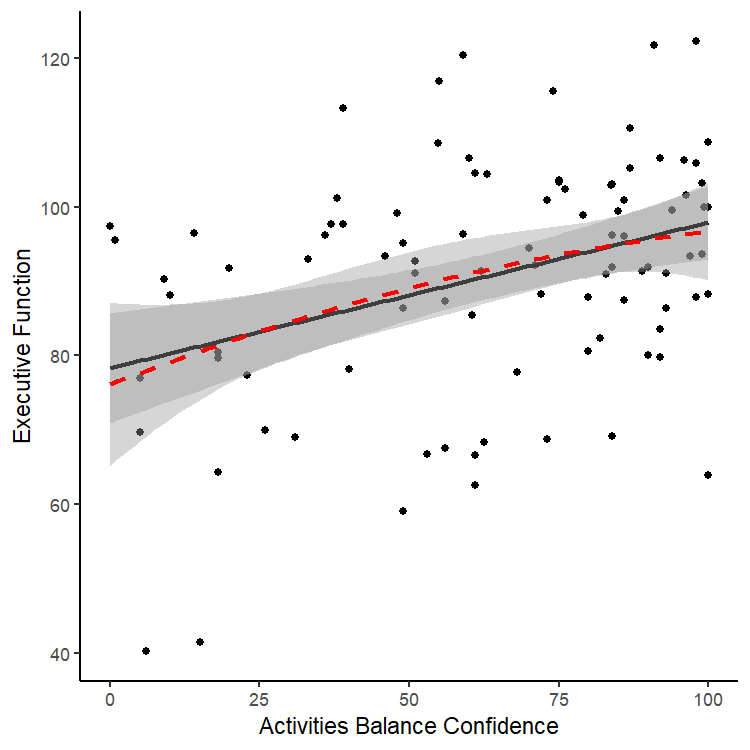


Supplemental Table 5: Characteristics of individuals who were and were not missing QOL data.

| **Variable**^1^ | **Non-missing**  N = 74^1^ | **Missing**  N = 21^1^ | **p-value**^1^ |
| --- | --- | --- | --- |
| Age (years) | 72.0 ± 7.8 | 69.7 ± 9.3 | 0.3 |
| Gender |  |  | 0.4 |
| F | 29 (39%) | 6 (29%) |  |
| M | 45 (61%) | 15 (71%) |  |
| Activities of Balance Confidence (%) | 62.7 ± 28.7 | 65.7 ± 30.8 | 0.6 |
| Gait Velocity (cm/sec) | 81.0 ± 26.9 | 89.9 ± 30.3 | 0.072 |
| Global Cognitive Score² | 92.3 ± 13.6 | 93.6 ± 14.7 | 0.5 |
| Executive Function Score² | 90.5 ± 15.0 | 91.8 ± 19.1 | 0.7 |
| Missing | 1 | 0 |  |
| Attention Score² | 91.0 ± 20.3 | 92.3 ± 18.8 | >0.9 |
| Missing | 8 | 1 |  |
| Quality of Life³ | 66.4 ± 19.1 | NA ± NA |  |
| Missing | 0 | 21 |  |
| Hoehn & Yahr |  |  | >0.9 |
| 1 | 4 (5.4%) | 2 (9.5%) |  |
| 1.5 | 1 (1.4%) | 0 (0%) |  |
| 2 | 21 (28%) | 6 (29%) |  |
| 2.5 | 4 (5.4%) | 1 (4.8%) |  |
| 3 | 38 (51%) | 10 (48%) |  |
| 4 | 6 (8.1%) | 2 (9.5%) |  |
| ^1^Mean ± SD or %; ²NeuroTrax; ³NeuroQOL | | | |
